# Feasibility of T2-Weighted MRI Radiomics for Initial Risk Stratification in Pediatric Neuroblastoma

**DOI:** 10.3390/children13040450

**Published:** 2026-03-26

**Authors:** Annalisa Tondo, Irene Ferri, Mattia Biavati, Federica Carra, Irene Trambusti, Andrea Di Cataldo, Maurizio Aricò, Lorenzo Lasagni, Ubaldo Bongini, Margherita Trinci, Francesca Fierro, Anna Perrone

**Affiliations:** 1 Oncology, Hematology, and Stem Cell Transplantation, Meyer Children’s Hospital IRCCS, 50139 Florence, Italy; irene.ferri@edu.unife.it (I.F.); mattia.biavati@unipi.it (M.B.); federica.carra@meyer.it (F.C.); irene.trambusti@meyer.it (I.T.); 2 Pediatric Hemato-Oncology Unit, Department of Clinical and Experimental Medicine, Policlinico Rodolico-San Marco, University of Catania, 95123 Catania, Italy; adicata@unict.it; 3 Interdisciplinary Department of Medicine, Aldo Moro University of Bari, 70124 Bari, Italy; maurizio.arico55@gmail.com; 4 Department of Physics and Astronomy, University of Florence, 50019 Florence, Italy; lorenzo.lasagni@meyer.it; 5 Department of Pediatric Radiology, Meyer Children’s Hospital IRCCS, 50139 Florence, Italy; ubaldo.bongini@meyer.it (U.B.); margherita.trinci@meyer.it (M.T.); francesca.fierro@meyer.it (F.F.); anna.perrone@meyer.it (A.P.)

**Keywords:** neuroblastoma, pediatric oncology, radiomics, magnetic resonance imaging, risk stratification, machine learning, imaging biomarkers

## Abstract

**Highlights:**

**What are the main findings?**

- Radiomics extracted from routine T2-weighted magnetic resonance imaging (MRI) enabled initial risk stratification in pediatric neuroblastoma (NB), achieving a test accuracy of 77.8% (specificity 85.7%).
- Radiomic classification showed good agreement with currently adopted clinical risk stratification systems.

**What are the implications of the main findings?**

- T2-weighted MRI radiomics may represent a feasible, noninvasive imaging biomarker to support early risk assessment at diagnosis.
- These preliminary findings warrant prospective, multicenter validation to better define the incremental value of radiomics alongside established clinical and molecular risk classification frameworks in NB.

**Abstract:**

**Purpose**: The purpose of this study was to evaluate the feasibility of magnetic resonance imaging (MRI)-based radiomics derived from routine T2-weighted imaging for initial risk stratification in pediatric neuroblastoma (NB) and to explore its potential role as a noninvasive adjunct to established clinical and molecular classification systems. **Methods**: In this retrospective, single-center pilot study, 45 children with newly diagnosed NB (2015–2024) were analyzed. Primary tumors were manually segmented on baseline axial T2-weighted MRI. A total of 107 Image Biomarker Standardization Initiative (IBSI)-compliant radiomic features were extracted. Supervised machine learning classifiers (Random Forest, XGBoost) and dimensionality reduction approaches (principal component analysis [PCA], linear discriminant analysis [LDA]) combined with K-means clustering were evaluated. Model performance was assessed using stratified cross-validation and an independent test set. Reporting adhered to the Checklist for Evaluation of Radiomics Research (CLEAR). **Results**: Fifteen patients (33%) were classified as high-risk (HR) and 30 (67%) as non-high-risk (NHR) according to International Neuroblastoma Risk Group (INRG) criteria. The highest classification performance was achieved using LDA followed by K-means clustering, with a test accuracy of 77.8%, sensitivity of 64.7%, and specificity of 85.7%. Radiomic classification agreed with conventional risk stratification in 77.8% of cases. The analysis relied exclusively on T2-weighted imaging, supporting workflow feasibility without requiring contrast administration or advanced MRI sequences. **Conclusions**: In this single-center pilot study, T2-weighted MRI radiomics demonstrated feasibility for noninvasive initial risk stratification in pediatric NB. Although limited by sample size and the lack of external validation, these findings support further multicenter investigations of radiomics as an adjunctive imaging biomarker during early diagnostic evaluation.

## 1. Introduction

Neuroblastoma (NB) is the most common extracranial solid malignancy of childhood, accounting for approximately 8–10% of pediatric cancers and up to 15% of cancer-related mortality in children [1,2]. The clinical behavior of NB is highly heterogeneous, ranging from spontaneous regression to aggressive metastatic disease, making accurate risk stratification at diagnosis essential for guiding treatment intensity and optimizing outcomes.

Current risk classification systems, including the International Neuroblastoma Risk Group (INRG) framework, integrate clinical variables, histopathology, and molecular features such as MYCN amplification and segmental chromosomal aberrations (SCAs) [3]. Although highly effective, these systems depend on invasive tissue sampling and specialized molecular analyses, which may be time-consuming or delayed during the early diagnostic phase. In selected clinical scenarios, such as large, surgically challenging tumors, definitive molecular risk assignment may not be immediately available at presentation.

Radiomics has emerged as a quantitative imaging approach capable of extracting high-dimensional features that describe tumor morphology, texture, and signal heterogeneity from standard medical images [4]. When applied to magnetic resonance imaging (MRI), radiomics offers a noninvasive means of exploring tumor phenotype and underlying biology. In pediatric NB, early radiomic and radiogenomic studies have reported associations between imaging-derived features and molecular markers, including MYCN amplification, disease stage, and clinical risk group [5,6,7,8]. However, these studies are largely retrospective, based on small cohorts, and characterized by heterogeneity in imaging protocols and analytical methods, limiting reproducibility and clinical translation.

A key unmet need therefore remains for radiomic approaches that are feasible, standardized, clinically translatable, and compatible with routine diagnostic workflows. Many prior studies relied on contrast-enhanced or diffusion-weighted MRI sequences, which may be more susceptible to protocol variability and intrascanner differences, potentially limiting reproducibility. In contrast, T2-weighted MRI sequences are routinely obtained at diagnosis and robust across scanners and do not require contrast administration, making them particularly suitable for reproducible radiomic analysis in pediatric populations. The primary aim of this pilot study was to evaluate the feasibility of MRI-based radiomics derived exclusively from T2-weighted sequences for initial risk stratification in pediatric NB. By adhering to standardized feature extraction and reporting recommendations, aimed at improving reproducibility, including the Checklist for Evaluation of Radiomics Research (CLEAR) [9], we sought to explore whether radiomic patterns could recapitulate established clinical risk groups using a minimally complex and workflow-compatible approach. This study is intended as a hypothesis-generating step toward the future multicenter validation and integration of imaging biomarkers into early diagnostic decision-making.

## 2. Materials and Methods

### 2.1. Study Design and Patient Population

This retrospective, single-center feasibility study included children with pathologically confirmed NB diagnosed between January 2015 and December 2024 at Meyer Children’s Hospital IRCCS (Florence, Italy). Patients were classified as high-risk (HR) or non-high-risk (NHR) according to the INRG classification system [3]. Clinical, demographic, and laboratory data, including age at diagnosis, disease stage, histology, MYCN status, SCAs, and urinary vanillylmandelic acid (VMA) levels, were retrieved from medical records.

The initial cohort comprised 66 pediatric patients with pathologically confirmed NB diagnosed between January 2015 and December 2024. Of these, 21 patients were excluded for the following reasons: no baseline MRI available at the time of initial diagnosis (*n* = 14), MRI performed after the commencement of treatment (*n* = 4), and non-diagnostic image quality precluding reliable segmentation (*n* = 3). The final study cohort therefore consisted of 45 patients. Inclusion criteria were: (1) pathologically confirmed NB; (2) the availability of baseline axial T2-weighted MRI performed prior to any treatment; and (3) adequate image quality for radiomic analysis. Exclusion criteria were: (1) the absence of diagnostic-quality baseline MRI; (2) imaging acquired after the initiation of therapy; and (3) non-diagnostic image quality. Informed consent was waived due to the retrospective analysis of anonymized data.

### 2.2. MRI Acquisition

Baseline MRI examinations were performed as part of routine clinical care using either 1.5 T or 3 T scanners (Achieva and Ingenia platforms, Philips Medical Systems, Best, The Netherlands), with standard pediatric body coils. Axial T2-weighted fast spin echo sequences were acquired with the following representative parameters: repetition time 2051–3000 ms, echo time 100 ms, slice thickness 3–4 mm, no interslice gap, field of view 250–380 mm, and acquisition matrix 200 × 200 (whole body MRI)/320 × 320 (abdomen MRI). Among the 45 included patients, 28 (62%) were examined on a 1.5 T scanner and 17 (38%) on a 3 T scanner. To mitigate potential field strength-related signal variability, all radiomic features were normalized using Z-score standardization prior to analysis, a strategy supported by prior studies addressing MRI signal heterogeneity in radiomic workflows. For the purposes of this study, only axial T2-weighted images were analyzed. This sequence was selected because it is universally acquired at diagnosis, does not require contrast administration, and is less susceptible to protocol variability compared with advanced functional imaging.

### 2.3. Tumor Segmentation

**Figure 1:**
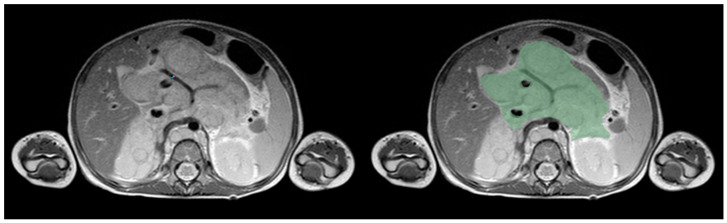
Example of axial MRI slice with tumor segmentation. Example of manual tumor segmentation on axial T2-weighted MRI. Primary tumor was segmented slice by slice to generate three-dimensional volume of interest. Abbreviations: MRI, magnetic resonance imaging.

Primary tumors were manually segmented on axial T2-weighted images using 3D Slicer software (version 4.11) [10]. Segmentation was performed by a pediatric radiologist with nine years of experience in oncologic imaging (F.F.) and independently reviewed by a second senior pediatric radiologist with sixteen years of experience in pediatric oncology imaging, specifically with NB (A.P.), to ensure anatomical accuracy. In cases of disagreement, consensus was reached through joint review. Segmentations were performed slice by slice to generate three-dimensional volumes of interest encompassing the entire primary tumor. An example of tumor segmentation is shown in Figure 1.

### 2.4. Radiomic Feature Extraction and Preprocessing

Radiomic feature extraction was performed using the PyRadiomics library (version 3.0.1) [11]. A total of 107 radiomic features were extracted from each tumor volume, including first-order statistics, shape-based features, and texture features derived from gray-level co-occurrence, run length, size zone, dependence, and neighboring gray-tone difference matrices. Feature definitions and extraction parameters were compliant with Image Biomarker Standardization Initiative (IBSI) recommendations to ensure methodological reproducibility and transparency [12].

Prior to analysis, all radiomic features were normalized using Z-score standardization to reduce scale-related bias. Critically, normalization parameters were calculated exclusively on training data during cross-validation and applied to validation and test sets to prevent data leakage. No outcome-based feature selection was applied before dimensionality reduction to minimize the risk of information leakage.

### 2.5. Dimensionality Reduction and Machine Learning Strategy

Given the limited sample size and exploratory nature of this feasibility study, multiple analytical approaches were evaluated with an emphasis on minimizing model complexity. Supervised machine learning classifiers, including Random Forest [13] and XGBoost [14], were implemented to assess the direct prediction of risk group based on radiomic features.

In parallel, dimensionality reduction techniques were applied to project high-dimensional radiomic data into lower-dimensional feature spaces. Principal component analysis (PCA) was used as an unsupervised method to capture variance within the dataset [15], while linear discriminant analysis (LDA) was used as a supervised method to project data into a lower-dimensional space maximizing separation between the HR and NHR groups [16]. Following dimensionality reduction, K-means clustering (k = 2) was applied to the transformed data [17].

We emphasize that the clustering analyses are not intended as unsupervised discoveries of novel risk classes. Rather, they serve two specific exploratory purposes: (1) to assess whether radiomic features contain sufficient discriminatory information to separate patients in a manner consistent with clinical labels when projected into lower-dimensional spaces and (2) to evaluate the robustness of this separation using a method (K-means) that makes no assumptions about class boundaries. The subsequent calculation of classification metrics against clinical labels is therefore a post hoc descriptive evaluation of cluster agreement, not a validation of a predictive model. This approach is conceptually analogous to visualizing class separability in a reduced feature space.

Critically, all dimensionality reduction techniques (PCA and LDA) were applied exclusively within the training data during cross-validation, with the learned transformations then applied to validation and test sets to prevent information leakage.

This hybrid approach was deliberately chosen to explore the intrinsic radiomic structure while limiting overfitting risk in a small dataset and to assess agreement between data-driven clustering and clinically defined risk categories rather than to develop a definitive predictive model.

### 2.6. Model Training and Validation

The dataset was partitioned using stratified random sampling, with 80% of patients (*n* = 36) allocated to the training set and 20% (*n* = 9) reserved as an independent held-out test set. Stratification ensured the proportional representation of HR and NHR patients in both sets. The test set was held out prior to all model training and hyperparameter optimization steps and was used only for final performance evaluation. 

All model development, including preprocessing, feature normalization, dimensionality reduction, and hyperparameter tuning, was performed exclusively within the training set using five-fold stratified cross-validation. 

For supervised models, hyperparameter tuning was performed using grid search within the cross-validation framework. The Random Forest hyperparameters explored included the number of trees (100–500), maximum depth (5–20), and minimum samples split (2–10). XGBoost parameters included the learning rate (0.01–0.3), max depth (3–9), and number of estimators (50–200). The final models were retrained on the full training set using the optimal parameters identified through cross-validation before test set evaluation.

Performance metrics included accuracy, sensitivity, specificity, precision, recall, and F1-score. For the clustering approaches, cluster-to-class label assignment was optimized using a contingency matrix. All analyses were conducted using Python (version 3.9) with the scikit-learn library (version 1.0.2) [18]. A fixed random seed (42) was applied to ensure reproducibility.

### 2.7. Reporting Standards and Reproducibility

This study was conducted and reported in accordance with the CLEAR guidelines [9]. The full imaging workflow, preprocessing steps, and analytical pipeline were documented to promote transparency. Analysis scripts and parameter settings are available from the corresponding author upon reasonable request.

## 3. Results

### 3.1. Patient Characteristics

A total of 45 patients were included in the analysis, comprising 21 males and 24 females, with a median age at diagnosis of 32 months (range: 0.6–184 months). According to the INRG, 15 patients (33%) were classified as HR and 30 (67%) as NHR. Baseline clinical and biological characteristics, including INRG stage, histology, MYCN status, SCAs, and urinary vanillylmandelic acid levels, are summarized in Table 1.

MYCN amplification was detected in two patients, while SCAs were reported in 11. Molecular data were incomplete in a subset of patients, reflecting real-world diagnostic availability in a retrospective cohort.

### 3.2. Performance of Supervised Classification Models

Among supervised machine learning approaches, XGBoost demonstrated higher classification performance than Random Forest. XGBoost achieved a test accuracy of 66.7%, compared with 50.0% for Random Forest, as shown in Table 2. Nevertheless, both supervised models showed only moderate performance, with discrepancies between the cross-validation and test results, underscoring the limitations of direct supervised prediction in a small, heterogeneous dataset.

### 3.3. Dimensionality Reduction and Clustering Analysis

Dimensionality reduction followed by clustering demonstrated higher agreement with clinical risk stratification than supervised classification alone. PCA combined with K-means achieved a test accuracy of 66.7%. The highest performance was observed using LDA followed by K-means clustering, with a test accuracy of 77.8%, sensitivity of 64.7%, and specificity of 85.7%, as shown in Table 2. The high cross-validation accuracy for LDA+K-means (97.2%) relative to test accuracy (77.8%) suggests some degree of overfitting even with this approach, despite dimensionality reduction.

### 3.4. Confusion Matrix and Agreement with Clinical Risk Groups

**Figure 2:**
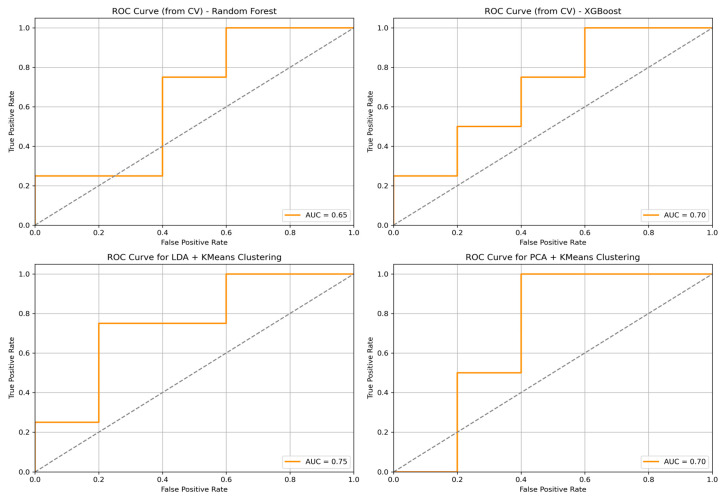
Receiver operating characteristic (ROC) curve for all algorithms. ROC curve illustrating performance of various approaches in classifying high-risk (HR) versus non-high-risk (NHR) patients. Abbreviations: HR, high-risk; NHR, non-high-risk; ROC, receiver operating characteristic.

**Figure 3:**
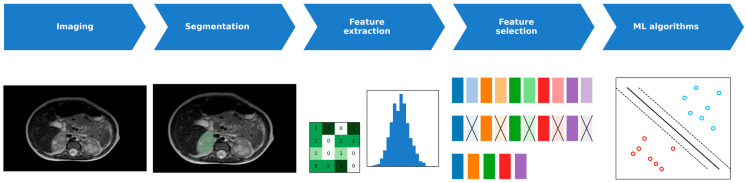
Workflow diagram of radiomic feature extraction and classification. Radiomic pipeline including segmentation, feature extraction, and classification. Abbreviations: ML, machine learning.

The confusion matrix for the LDA plus K-means model is reported in Table 3. Eleven of 15 clinically defined HR patients were correctly identified, while four HR patients were misclassified as NHR. Among NHR patients, 24 of 30 were correctly classified, with six misclassified as HR. The resulting negative predictive value (NPV) was 80.0%, indicating greater reliability in identifying patients unlikely to belong to the HR group. Receiver operating characteristic curves illustrating the performance of the evaluated approaches are shown in Figure 2, while the overall analytical workflow is summarized in Figure 3.

### 3.5. Summary of Findings

Overall, radiomic analysis based exclusively on T2-weighted MRI demonstrated the ability to distinguish HR from NHR NB patients with moderate-to-good agreement with established clinical risk stratification. Performance varied across analytical strategies, with dimensionality reduction-based approaches showing superior performance than the supervised classifiers evaluated in this study.

## 4. Discussion

In this single-center pilot study, we evaluated the feasibility of MRI-based radiomics derived exclusively from routine T2-weighted imaging for initial risk stratification in pediatric NB. Our findings demonstrate that radiomic features extracted from standard MRI can achieve moderate-to-good agreement with established INRG classification, supporting the potential role of radiomics as a noninvasive adjunct during early diagnostic evaluation. However, these results must be interpreted with caution given the methodological limitations inherent to this exploratory study.

The best overall performance was observed using LDA followed by K-means clustering, with cluster assignments subsequently compared with the reference INRG risk groups, achieving a test accuracy of 77.8% and a specificity of 85.7% for identifying NHR patients. Notably, dimensionality reduction-based approaches outperformed direct supervised classifiers, suggesting that radiomic patterns may capture imaging phenotypes associated with clinical risk categories. The higher specificity for NHR identification is clinically interesting, as it suggests that T2-based radiomics may be particularly useful for identifying patients unlikely to belong to the HR group during the early diagnostic phase. In practice, such information could support preliminary multidisciplinary discussions while awaiting definitive histopathologic and molecular results.

However, several important methodological limitations must be acknowledged. The most significant is the small sample size (45 patients, 15 HR) relative to the number of extracted features (107). This high-dimensional problem substantially increases the risk of overfitting and limits model stability. The observed discrepancy between cross-validation and test performance, particularly for supervised models, reflects this challenge. Even the LDA + K-means approach, despite dimensionality reduction, showed a substantial drop from cross-validation (97.2%) to test accuracy (77.8%), suggesting residual overfitting despite dimensionality reduction. We emphasize that this study is hypothesis-generating, not confirmatory, and the reported performance metrics should be viewed as preliminary estimates requiring validation in independent cohorts.

Methodological rigor and transparency were prioritized in this study through adherence to the CLEAR and compliance with IBSI recommendations for feature extraction. The use of standardized software tools and explicit reporting of analytical steps enhances reproducibility and facilitates comparison with future studies. Nevertheless, several limitations must be acknowledged. The absence of external validation represents the most important limitation and the primary barrier to clinical translation, as the results may not generalize to other centers with different MRI hardware, acquisition protocols, or patient populations. External validation, ideally in a prospective, multicenter cohort with harmonized imaging protocols, is an absolute prerequisite before any clinical application can be considered. Additional limitations include variability in MRI acquisition parameters and field strengths (1.5 T vs. 3 T), which may affect radiomic feature stability and the retrospective design with inherent selection bias.

Our results are broadly consistent with prior pilot studies exploring radiomics in NB [5,6,7,8], which have reported associations between imaging features and molecular markers or risk groups. However, direct comparison is difficult due to heterogeneity in imaging sequences, feature extraction methods, and analytical approaches. By focusing exclusively on T2-weighted imaging and adhering to IBSI and CLEAR recommendations, we prioritized methodological transparency and reproducibility.

Importantly, radiomic analysis is not intended to replace established classifications but rather to complement existing clinical and biologic information with quantitative imaging biomarkers that capture tumor heterogeneity beyond visual assessment.

Despite these limitations, the present study reflects real-world feasibility in a pediatric oncology setting and highlights the potential of minimally complex, MRI-based radiomic approaches. Future research should focus on multicenter validation, the harmonization of imaging protocols, and the integration of radiomic features with clinical and molecular variables to improve predictive performance. The incorporation of semi-automated segmentation and prospective study designs may further enhance clinical translatability.

## 5. Conclusions

MRI-based radiomics derived exclusively from routine T2-weighted imaging appears feasible for noninvasive initial risk stratification in pediatric NB. In this single-center pilot study, radiomic patterns demonstrated moderate-to-good agreement with established classification, supporting the ability of standard MRI to capture clinically relevant tumor heterogeneity.

Although limited by sample size and the lack of external validation, these findings highlight the potential of workflow-compatible radiomics as an adjunctive imaging biomarker during early diagnostic evaluation. Future multicenter studies incorporating harmonized imaging protocols and integrated clinical and molecular data are warranted to validate and extend these preliminary observations.

## Figures and Tables

**Table 1:** Presenting features of 45 patients with newly diagnosed neuroblastoma.

Characteristic	Value
**Age (months**)	median 32 (range: 0.6–184)
**Gender**	
male	21
female	24
**INRG stage**	
L1	19
L2	12
M	12
MS	2
**Risk group**	
HR	15
NHR	30
**MYCN**	
amplified	2
not amplified	30
missing data	13
**SCAs**	
present	11
absent	9
missing data	25
**Histology**	
undifferentiated	13
intermixed ganglioneuroblastoma	6
poorly differentiated	15
nodular ganglioneuroblastoma	7
missing data	4
**Urinary VMA**	
normal	9
elevated	35
not available	1

Baseline clinical and biological characteristics of 45 patients with newly diagnosed neuroblastoma. Abbreviations: INRG, International Neuroblastoma Risk Group; HR, high-risk; NHR, non-high-risk; SCAs, segmental chromosomal aberrations; VMA, urinary vanillylmandelic acid.

**Table 2:** Performance metrics of supervised and unsupervised classification algorithms.

Algorithm	CV Accuracy	Test Accuracy	Precision	Recall	F1-Score
Random Forest	69.3%	50.0%	0.48	0.48	0.48
XGBoost	71.4%	66.7%	0.79	0.7	0.65
PCA + K-Means	61.1%	66.7%	0.68	0.68	0.67
LDA + K-Means	97.2%	77.8%	0.65	0.73	0.77

Performance metrics of supervised and dimensionality reduction-based classification approaches. Abbreviations: CV, cross-validation; PCA, principal component analysis; LDA, linear discriminant analysis.

**Table 3:** Performance of radiomic model in classifying patients with high-risk versus non-high-risk neuroblastoma.

	Predicted HR (Model Output: HR)	Predicted NHR (Model Output: NHR)
Actual HR (by INRG)	11	6
Actual NHR (by INRG)	4	24

Confusion matrix for LDA + K-means model. Rows represent actual (clinically defined) INRG risk group; columns represent predicted risk group assigned by radiomic model. Values represent number of patients. Abbreviations: HR, high-risk; NHR, non-high-risk.

## Data Availability

The dataset generated for this study is not publicly available, and imaging data are currently undergoing anonymization. The data may be made available upon reasonable request to the corresponding author.

## Abbreviations

CT	Computed Tomography
CV	Cross-Validation
CLEAR	Checklist for Evaluation of Radiomics Research
HR	High-Risk
IBSI	Image Biomarker Standardization Initiative
INRG	International Neuroblastoma Risk Group
LDA	Linear Discriminant Analysis
MRI	Magnetic Resonance Imaging
NPV	Negative Predictive Value
NB	Neuroblastoma
NHR	Non-High-Risk
PCA	Principal Component Analysis
ROC	Receiver Operating Characteristic
SCAs	Segmental Chromosomal Aberrations
VMA	Urinary Vanillylmandelic Acid
